# Rational Design of a Modality‐Specific Inhibitor of TRPM8 Channel against Oxaliplatin‐Induced Cold Allodynia

**DOI:** 10.1002/advs.202101717

**Published:** 2021-10-17

**Authors:** Aerziguli Aierken, Ya‐Kai Xie, Wenqi Dong, Abuliken Apaer, Jia‐Jia Lin, Zihan Zhao, Shilong Yang, Zhen‐Zhong Xu, Fan Yang

**Affiliations:** ^1^ Department of Biophysics Kidney Disease Center of the First Affiliated Hospital Zhejiang University School of Medicine Hangzhou Zhejiang Province 310058 China; ^2^ NHC and CAMS Key Laboratory of Medical Neurobiology MOE Frontier Science Center for Brain Research and Brain–Machine Integration School of Brain Science and Brain Medicine Zhejiang University Hangzhou Zhejiang 310058 China; ^3^ Department of Neurobiology and Department of Anesthesiology of First Affiliated Hospital Zhejiang University School of Medicine Hangzhou Zhejiang 310058 China; ^4^ College of Wildlife and Protected Area Northeast Forestry University Harbin 150040 China

**Keywords:** designed protein, ion channels, oxaliplatin‐induced cold allodynia, pain, TRPM8

## Abstract

Platinum‐based compounds in chemotherapy such as oxaliplatin often induce peripheral neuropathy and neuropathic pain such as cold allodynia in patients. Transient Receptor Potential Melastatin 8 (TRPM8) ion channel is a nociceptor critically involved in such pathological processes. Direct blockade of TRPM8 exhibits significant analgesic effects but also incurs severe side effects such as hypothermia. To selectively target TRPM8 channels against cold allodynia, a cyclic peptide DeC‐1.2 is de novo designed with the optimized hot‐spot centric approach. DeC‐1.2 modality specifically inhibited the ligand activation of TRPM8 but not the cold activation as measured in single‐channel patch clamp recordings. It is further demonstrated that DeC‐1.2 abolishes cold allodynia in oxaliplatin treated mice without altering body temperature, indicating DeC‐1.2 has the potential for further development as a novel analgesic against oxaliplatin‐induced neuropathic pain.

## Graphical abstract

To selectively target TRPM8 channels against cold allodynia, a cyclic peptide DeC‐1.2 is computationally designed with the optimized hot‐spot centric approach. It inhibits the ligand activation of TRPM8 but not the cold activation. DeC‐1.2 also abolishes cold allodynia in oxaliplatin treated mice without altering body temperature, indicating its potential as a novel analgesic against oxaliplatin‐induced neuropathic pain.

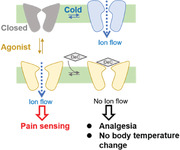

## 1 Introduction

Peripheral neuropathy is a common side effect of many platinum‐based compounds in chemotherapy, which limits the dosage of anticancer drugs and lowers the quality of life of patients taking these drugs.^[^ ^1^, ^2^ ^]^ For instance, oxaliplatin is commonly used as a first‐line chemotherapy for many tumors, such as colorectal^[^ ^3^ ^]^ and gastric^[^ ^4^ ^]^ cancers. However, oxaliplatin often rapidly induces acute neurotoxicity in up to 89% of patients taking this drug,^[^ ^5^ ^]^ hallmarked by cold allodynia in the hands and feet of patients where a normally cool temperature is detected as extreme coldness to trigger pain sensation. Though most of the chemotherapy‐induced neuropathy and neuropathic pain symptoms, including the cold allodynia induced by oxaliplatin, would improve upon drug withdraw, to date there is still no effective treatment targeting such neuropathy.

Transient receptor potential melastatin 8 (TRPM8) ion channel is critically involved in the pathogenesis of oxaliplatin‐induced cold allodynia. TRPM8 is a non‐selective cation channel highly expressed in nociceptive neurons. As this channel is activated at temperature lower than 28 °C,^[^ ^6^, ^7^ ^]^ it is a sensor for coolness in mammalians.^[^ ^8^, ^9^ ^]^ Moreover, TRPM8 is a polymodal receptor also activated by chemical ligands such as menthol and icilin,^[^ ^10^ ^]^ as well as transmembrane depolarization.^[^ ^11^ ^]^ In the mouse mode of oxaliplatin‐induced cold allodynia, the expression level of TRPM8 channel in the nociceptive dorsal root ganglion (DRG) neurons was significantly elevated. More importantly, in TRPM8 knock‐out mice, the oxaliplatin‐induced cold allodynia was also abolished.^[^ ^12^ ^]^ Therefore, TRPM8 channel is a validated target for oxaliplatin‐induced cold allodynia.

Though there is no peptide toxin targeting TRPM8 channel identified from venomous animals, many small molecules have been screened and developed as TRPM8 inhibitors. Most of these inhibitors non‐discriminatively suppress all modes of TRPM8 activation, so they often caused hypothermia and changes in acute cold sensation in patients and thus failures in clinical trials.^[^ ^13^ ^]^ To overcome such limitations in pan‐mode inhibitors, one of the effective strategies is to develop modality‐specific TRPM8 inhibitor that spares cold activation of this channel.

**Figure 1:**
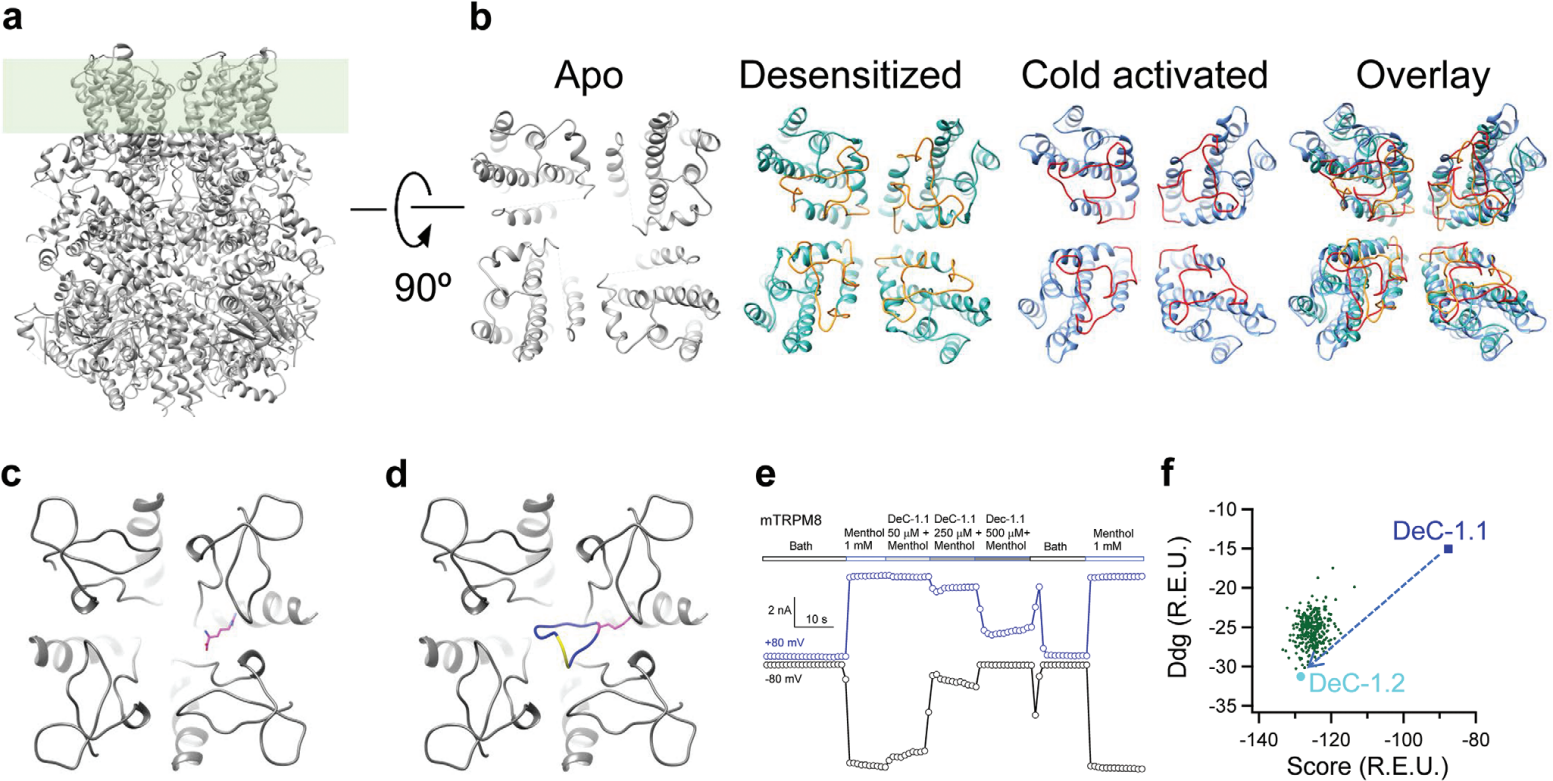
Rational design of cyclic peptides DeC1. a) Sideview of Cryo‐EM structure of TRPM8 in the apo state (PDB ID: 6O6A). b) Top view of TRPM8 outer pore region in the apo, desensitized (PDB ID: 6O77), and our computational model of cold activated state. c) An arginine (colored in purple) stably docked to the outer pore of TRPM8 as the optimal hotspot. d) The designed peptide DeC‐1.1 with two cysteine residues forming a disulfide bond (colored in yellow) bound to outer pore of TRPM8. e) Representative whole‐cell recording of DeC‐1.1 inhibiting menthol activation of mouse TRPM8 channel. f) In silico affinity maturation of DeC‐1.1 improving both the binding energy (Rosetta energy term ddg) and peptide stability (Rosetta energy term score) to get DeC‐1.2.

To develop a modality‐specific TRPM8 inhibitor, we took advantage of the recent breakthrough in TRPM8 channel structure biology. High‐resolution structures of TRPM8 channel have been determined by cryo‐EM in the apo, ligand bounded and desensitized states.^[^ ^14^, ^15^, ^16^ ^]^ The outer pore (selectivity filter and the linker to the beginning of S6) region of TRPM8 channel exhibits considerable structural flexibility, where this region is not resolved in the apo state (**Figure** **1a,b**). The conformation of outer pore region has been resolved in the desensitized state (Figure 1b, highlighted in orange; PDB ID: 6O77), but it is distinct to the model of TRPM8 we proposed before (Figure 1b, highlighted in red), where unnatural amino acid fluorescence information was incorporated in computational modeling to derive a potential structural model of TRPM8 channel pore in its cold activated state (Figure 1b).^[^ ^17^ ^]^ Based on such structural information, we hypothesized that as the outer pore region of TRPM8 shows state‐dependent conformational rearrangements. Therefore, it is plausible to achieve the modality‐specific inhibition by designing a modulator that preferentially binds to the outer pore region in the apo state but not the cold activated state.

In this study, we first computationally designed a cyclic peptide targeting the homology model of TRPM8 channel in the apo state using the hotspot‐centric protein design. This design approach has been applied to design protein binders to hemagglutinin in the influenza virus^[^ ^18^ ^]^ and binders to IgG.^[^ ^19^ ^]^ In particular, using this approach we have successfully designed peptidic positive allosteric modulators to the TRPV1 channel,^[^ ^20^ ^]^ which alleviate heat pain in rats. Here by patch‐clamp recording of TRPM8 channel expressed in HEK293 cells, we demonstrated that the designed peptide inhibited TRPM8 channel ligand activation with nanomolar IC_50_ and high selectivity among TRP channels, while it did not inhibit cold activation of the channel. We further observed that the designed peptide largely alleviated oxaliplatin‐induced cold allodynia in mice without significant perturbation of body temperature.

## 2 Results

### 2.1 Rational Design of the Cyclic Peptide DeC1

As we hypothesized that a modulator with preferential binding to the apo state of TRPM8 outer pore region is needed for modality‐specific inhibition of this channel, and the outer pore of TRPM8 in apo state has not been resolved in cryo‐EM structures,^[^ ^14^, ^16^ ^]^ we first computationally modeled this region based on the apo state structure of the closely related TRPM4 channel (PDB ID: 6BCL).^[^ ^21^ ^]^ We then rationally design a peptide binder to this region to block the channel with our optimized hotspot‐centric approach (OHCA).^[^ ^20^ ^]^ Briefly, to find the optimal hotspot for binding, we first performed molecular docking of each of the single amino acids toward the outer pore region. We observed that an arginine can stably bind to the outer pore (Figure 1c, residue in purple). Using this arginine as the hotspot, we computationally fused candidate peptide scaffolds, which were selected from the PDB data bank based on several criteria (see the Experimental Section for details), with the docked arginine hotspot. We further designed the amino acid sequence of the fused scaffold peptide with computational mutagenesis to optimize its binding with the TRPM8 outer pore region (Figure 1d, peptide in blue). To increase stability of the designed peptide, we introduced two cysteine residues to form a disulfide bond that links the N‐ and C‐terminus of the peptide, so the peptide became cyclic in structure (Figure 1d, residues in yellow).

To test function of this initially designed cyclic peptide (DeC‐1.1), this peptide was first chemically synthesized, and then purified by HPLC and validated by mass spectrometry (Figure S1a,b, Supporting Information). In whole‐cell patch‐clamp recording, we observed that DeC‐1.1 successfully inhibited the menthol activation of TRPM8 channel in a concentration‐dependent manner (Figure 1e). However, DeC‐1.1 exhibited a high IC_50_ value of 132.8 ± 73.5 × 10^−6^ m. To optimize DeC‐1.1, we performed computational affinity maturation, where both the in silico binding energy and protein stability (reflected by the Rosetta energy term ddg and score, respectively) of DeC‐1.1 were improved (Figure 1f).

**Figure 2:**
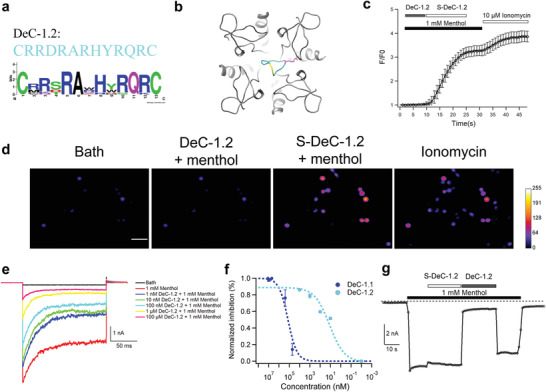
Functional validation of DeC‐1.2. a) Sequence logo of the amino acid sequence alignment among designed cyclic peptides with top ten binding energy. The height of a letter is proportional to the relative frequency of that residue at a particular site. The sequence of DeC‐1.2 is CRRDRARHYRQRC. b) DeC‐1.2 with the largest binding energy was shown in cyan. c) Average fluorescence response and d) representative calcium imaging of mTRPM8 in response to 1 × 10^−3^ m menthol, 500 × 10^−9^ m DeC‐1.2, 500 × 10^−9^ m S‐DeC‐1.2, and 10 × 10^−6^ m ionomycin, respectively (*n* = 20). Data in (d) are represented as average ± SEM. Scale bars, 100 × 10^−6^ m. e) Representative whole‐cell recording of DeC‐1.2 inhibiting menthol activation of mouse TRPM8 channel recorded at −80 mV. f) The concentration–response curves of DeC‐1.1 and DeC‐1.2 were measured with whole‐cell patch‐clamp recordings (*n* = 3). g) Representative whole‐cell current response of mTRPM8 to the scrambled peptide (S‐DeC‐1.2) and DeC‐1.2 (both at 500 × 10^−9^ m). The holding potential was −80 mV.

The designs with top ten binding energy from computational affinity maturation exhibited high sequence convergence (**Figure** **2a**), so we chose the design with the largest binding energy as the optimized design DeC‐1.2 (Figure 2a,b, peptide in cyan). The peptide of DeC‐1.2 was again chemically synthesized and purified in HPLC (Figure S1c, Supporting Information), which was then validated by mass spectrometry (Figure S1d, Supporting Information). We first performed calcium imaging to test the effect of DeC‐1.2 (Figure 2c,d). We observed that when co‐perfused with 1 × 10^−3^ m menthol, DeC‐1.2 blocked the activation of TRPM8 by menthol so that no increase in fluorescence occurred. When a scrambled peptide of DeC‐1.2 named S‐DeC‐1.2 (Table S2, Supporting Information) was co‐perfused with menthol, it did not block TRPM8 activation so that there was large increase in fluorescence because of calcium ion influx (Figure 2c,d). Furthermore, in patch‐clamp recordings DeC‐1.2 significantly inhibited the menthol‐induced channel activation (Figure 2e) with a much‐lowered IC_50_ value of 4.5 ± 3.0 × 10^−9^ m (Figure 2f), while its scrambled peptide did not show current inhibition even at a concentration of 500 × 10^−9^ m (Figure 2g).

### 2.2 Modality‐ and Subunit‐Specific Inhibition of TRPM8 Activation by DeC‐1.2

**Figure 3:**
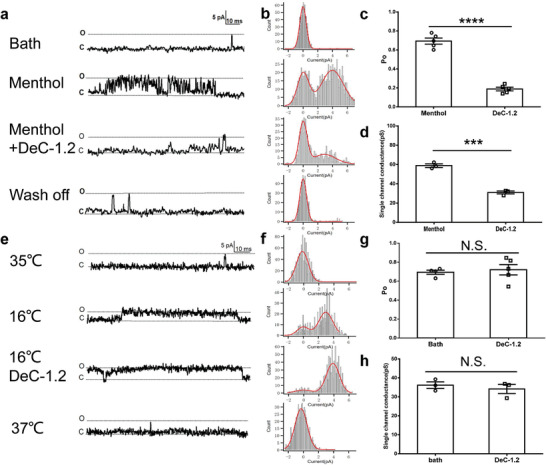
Modality‐specific inhibition of TRPM8 activation by DeC‐1.2. a) Representative single‐channel recordings of TRPM8 in outside‐out configuration at +80 mV. Saturating menthol (1 × 10^−3^ m) and the mixture of menthol (1 × 10^−3^ m) and DeC‐1.2 (100 × 10^−6^ m) were perfused to activate and inhibit the channel, respectively. These four representative recordings were from the same membrane patch. b) All‐point histograms of the representative single‐channel recordings shown in (a). Histograms were fitted to a double Gauss function (solid line in red), where the difference in two fitted peak values was used to calculate the single‐channel conductance. c) Open probability of TRPM8 was significantly decreased by DeC‐1.2 (100 × 10^−6^ m) in the presence of saturating menthol (1 × 10^−3^ m). Data were shown as Mean ± SEM of five cells for each group. **** indicates *p* < 0.0001. d) Single‐channel conductance of TRPM8 was significantly decreased when DeC‐1.2(100 × 10^−6^ m) was applied in the presence of saturating menthol (1 × 10^−3^ m). Data were shown as Mean ± SEM of three cells for each group. *** indicates *p* < 0.001. e) Representative single‐channel traces of TRPM8 cold activation recorded from inside‐out patches at +80 mV with or without DeC‐1.2 in the pipette solution (100 × 10^−6^ m). f) All‐point histograms of the representative single‐channel recordings shown in (e). Histograms were fitted to a double Gauss function. g) Open probability of TRPM8 was not significantly changed when DeC‐1.2 (100 × 10^−6^ m) was applied at 16 °C. Data were shown as Mean ± SEM of five cells for each group. N.S. indicates No significance. h) Single‐channel conductance of TRPM8 which showed no significant changes when DeC‐1.2(100 × 10^−6^ m) was applied at 16 °C. Data were shown as Mean ± SEM of three cells for each group. N.S. indicates No significance.

As DeC‐1.2 inhibited menthol activation of TRPM8 with high affinity, we next investigated whether this peptide showed modality‐specific inhibition as we designed. We performed single‐channel patch‐clamp recording, which allowed us to measure both the open probability and conductance of TRPM8 channel. In agreement with our previous study, the maximum open probability of TRPM8 activation by saturating concentration of menthol (1 × 10^−3^ m) was about 70% (**Figure** **3a–c**).^[^ ^10^ ^]^ When DeC‐1.2 (100 × 10^−6^ m) was applied with the saturated concentration of menthol, the open probability was significantly reduced to 18.83 ± 1.92% (n = 5) (Figure 3c). Moreover, we observed that the single‐channel conductance of TRPM8 was also decreased by DeC‐1.2 from 58.83 ± 1.85 pS (*n* = 3) to 30.97 ± 1.16 pS (*n* = 3) (Figure 3b,d), suggesting that DeC‐1.2 acted as a pore blocker to interfere the ion permeation in TRPM8.

We further examined whether DeC‐1.2 is sufficient to inhibit the cold activation of TRPM8. While cooling the membrane patch from 35 to 16 °C elicited TRPM8 single‐channel activities, the application of DeC‐1.2 (100 × 10^−6^ m) at 16 °C did not decrease the open probability (Figure 3e–g). Furthermore, though the single‐channel conductance of TRPM8 was decreased by cooling (36.16 ± 1.74 pS, *n* = 3) (Figure 3h), DeC‐1.2 did not further reduce the single‐channel conductance. Therefore, DeC‐1.2 inhibited TRPM8 channel in a modality‐specific manner as we designed.

**Figure 4:**
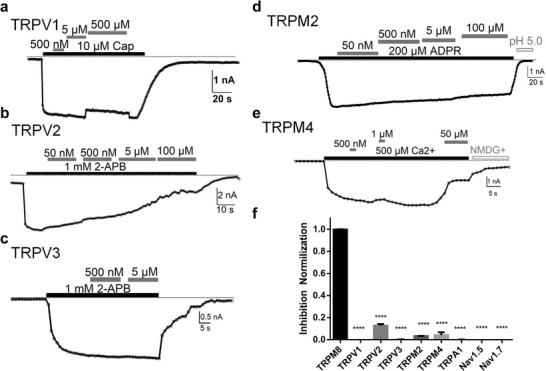
The subtype selectivity of DeC‐1.2. a–e) The representative whole‐cell current recording of DeC‐1.2 inhibition of TRPV1, TRPV2, TRPV3, TRPM2, and TRPM4 ligand activation, respectively. TRPM4‐K1045A mutant was used to remove PIP2 dependence. f) Comparison of normalized inhibition of DeC‐1.2 at 500 × 10^−9^ m against TRP channels and Nav channels in TRPV channel. The normalized inhibition of ion channels was determined by first measuring and calculating the fraction of current inhibition by 500 × 10^−9^ m DeC‐1.2 in each channel, and then the inhibition fraction for each channel was normalized to that of TRPM8. For each channel, *n* = 3, **** indicates *p* < 0.0001.

We further investigated the subtype selectivity of DeC‐1.2. TRPM8 channel belongs to the TRP channel super family,^[^ ^22^ ^]^ where the member channels share a similar topology as homotetramers with six transmembrane domains in each subunit. We found that while DeC‐1.2 inhibited TRPM8 channel with an IC_50_ value of 4.5 × 10^−9^ m (Figure 2f), more than 1000‐fold higher concentration of DeC‐1.2 (5 × 10^−6^ m) did not inhibit the ligand activation TRPV1 or TRPV3 (**Figure** **4a–c**). Although DeC‐1.2 inhibited TRPV2 channel to some extent, its inhibition on TRPV2 was far less efficient than on TRPM8, with an IC_50_ of about 10 × 10^−6^ m (Figure 4b and Table S1, Supporting Information). Among the TRP channels, TRPM2 is the closest homologue of TRPM8, but ligand activation of TRPM2 was not blocked by DeC‐1.2 up to 100 × 10^−6^ m (Figure 4d). When we designed DeC‐1.2, we used the structure of TRPM4 as the template for homology modeling of TRPM8 in the closed state, so DeC‐1.2 may exhibit higher affinity for TRPM4. However, we found that TRPM4 currents showed much lower sensitivity to DeC‐1.2, where 50 × 10^−6^ m DeC‐1.2 inhibited around half of TRPM4 currents (Figure 4e). We also tested DeC‐1.2 on TRPA1 and voltage‐gated sodium (Na_V_) channels and observed that none of the TRP channels and Na_V_ channels were inhibited by DeC‐1.2 (Table S1, Supporting Information). To illustrate the subtype selectivity of DeC‐1.2, we calculated the normalized inhibition of ion channels by first measuring and calculating the fraction of current inhibition by DeC‐1.2 in each channel, and then we normalized the inhibition fraction for each channel to that of TRPM8 (Figure 4f). Therefore, DeC‐1.2 not only exhibits high subtype selectivity, but also acts as a modality‐specific inhibitor to the ligand activation of TRPM8 channel.

**Figure 5:**
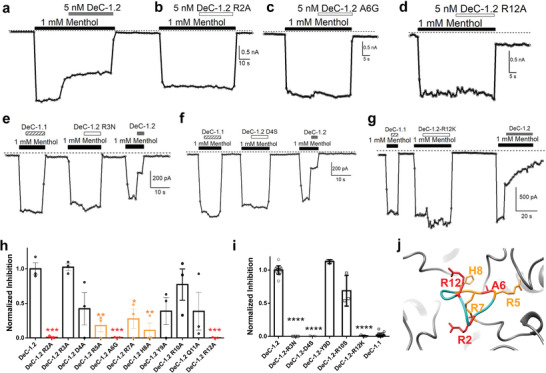
Critical residue(s) on DeC‐1.2 for its specific inhibition of TRPM8. a–d) Representative whole‐cell current recording of 5 × 10^−9^ m wild type DeC‐1.2, mutant R2A, A6G and R12A inhibition of TRPM8 ligand activation, respectively. e–g) Representative whole‐cell current recording of 5 × 10^−9^ m wild type DeC‐1.1, DeC‐1.2 and mutant R3N, D4S, and R12K inhibition of TRPM8 ligand activation, respectively. h) Normalized inhibition of the wild type DeC‐1.2 and its mutants against TRPM8 ligand activation at 5 × 10^−9^ m, *, **, and *** indicate *p* < 0.05, *p* < 0.01, and *p* < 0.001, respectively (*n* = 3). i) Normalized inhibition of the wild type DeC‐1.1, DeC‐1.2 and its mutants against TRPM8 ligand activation at 5 × 10^−9^ m, ** and **** indicate *p* < 0.01 and *p* < 0.0001, respectively. *n* = 3–18. j) The amino acid residues essential for TRPM8 inhibition as determined in alanine scan mapped onto the designed structure of DeC‐1.2. Residues eliminated or largely reduced channel inhibition were colored in red or orange, respectively.

We further performed alanine scanning to determine the key residue(s) on DeC‐1.2 for such an interaction with TRPM8. Each residue of DeC‐1.2 except for C1, A6, and C13 was mutated to alanine (A6 was mutated to glycine), respectively (Table S2, Supporting Information). These peptides with a single‐point mutation were chemically synthesized and purified as wild type DeC‐1.2 (Figure S2, Supporting Information). In whole‐cell patch‐clamp recordings, we observed that while the wild type DeC‐1.2 at 5 × 10^−9^ m decreased menthol‐induced TRPM8 current nearly by half (**Figure** **5a**), the same concentration of mutant R2A, A6G, and R12A barely inhibited TRPM8 channel (Figure 5a–d). The residue R5 serves as the hotspot in the design phase of DeC‐1.1, so when this hotspot was mutated, TRPM8 inhibition was largely and significantly abolished (Figure 5h).

As DeC‐1.2, which was computationally optimized based on DeC‐1.1, showed much lower IC_50_ than that of DeC‐1.1 (Figure 2f), we also investigated how the mutations introduced by computation affinity maturation contribute to the TRPM8 inhibition. We first compared the amino acid sequence of DeC‐1.1 and DeC‐1.2 (Table S3, Supporting Information) and mutated each of unconserved residue in DeC‐1.2 back to those in DeC‐1.1. We observed that among the six mutations introduced by optimization in DeC‐1.1 (N3R, S4D, A7R, D9Y, S10R, and K12R), reverse mutations R3N, D4S, and R12K in DeC‐1.2 largely abolished the inhibition of TRPM8 channel (Figure 5e–g,i). Therefore, we suggest that the charged residues introduced by our optimization process largely boosted the performance DeC‐1.2.

We further mapped the mutations that either eliminated or largely impaired TRPM8 inhibition found in the alanine scan on the designed structure of DeC‐1.2 (Figure 5j, residues in red and orange, respectively). Because most of the residues were designed to locate at the interface between DeC‐1.2 and the outer pore of TRPM8, the impaired TRPM8 inhibition by these mutations supported that DeC‐1.2 binds to TRPM8 as we designed.

### 2.3 DeC‐1.2 Inhibited Oxaliplatin‐Induced Cold Allodynia In Vivo without Affecting Body Temperature

**Figure 6:**
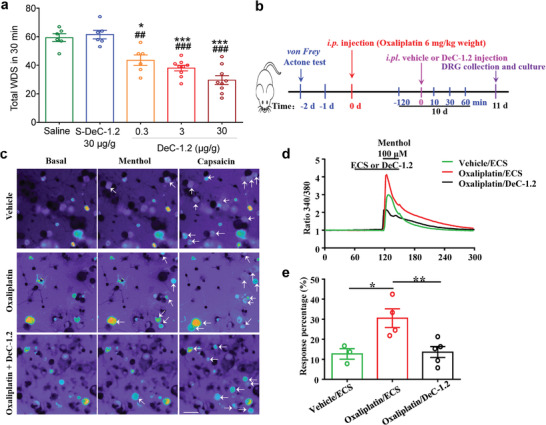
Effects of TRPM8 inhibition by DeC‐1.2 in vivo and ex vivo. a) DeC‐1.2 significantly and dose dependently reduced icilin induced wet dog shake (WDS) behavior in mice. Data were shown as Mean ± SEM of 6–9 animals for each group. *, **, and *** indicate *p* < 0.05, *p* < 0.01, and *p* < 0.001, respectively. b) Schematic illustration of the procedures to establish oxaliplatin‐induced cold allodynia in mice and in vivo tests of DeC‐1.2 against cold allodynia. c) Representative ratiometric calcium images of DRG neurons isolated from vehicle treated mice perfused with extracellular solution (Vehicle), oxaliplatin treated mice perfused with extracellular solution (Oxaliplatin) and oxaliplatin treated mice perfused with DeC‐1.2 (10 × 10^−6^ m) in extracellular solution (Oxaliplatin + DeC‐1.2). Increased calcium influx was observed upon TRPM8 channel activation by menthol application (100 × 10^−6^ m). Capsaicin (1 × 10^−6^ m) that activated TRPV1 channels in DRG neurons served as the positive control. The neurons responded to the agonists were pointed out by arrows in white. d) Representative fluorescent ratio traces in response to corresponding reagents in DRG neurons from vehicle treated and oxaliplatin treated mice. e) Percentage of menthol responding DRG neurons from vehicle treated mice and oxaliplatin treated mice. Total neurons for vehicle, oxaliplatin, and oxaliplatin + DeC‐1.2 were 229, 238, and 334, respectively. * and ** indicate *p* < 0.05 and *p* < 0.01, respectively. Each open circle represented one field of view for neuron counting.

Given that in vitro patch‐clamp recordings clearly demonstrated that DeC‐1.2 is a modality‐ and subtype‐specific inhibitor of TRPM8, we were prompted to investigate its function in vivo. The wet‐dog shake (WSD) behavior model induced by icilin, a potent agonist of TRPM8 channel, was used to evaluate the effect of DeC‐1.2 in mice (Figure 6a). We observed that intravenous injection of DeC‐1.2 dose dependently antagonized the WSD behaviors in mice, where a dosage as low as 0.3 µg g^−1^ (body weight) was enough to significantly reduce the behavior counts. The scrambled peptide S‐DeC‐1.2 did not affect the WDS behavior even at a high dosage of 30 µg g^−1^ (**Figure** **6a**). Such observations confirmed the in vivo effect of DeC‐1.2 upon TRPM8.

To further test whether DeC‐1.2 alleviates cold allodynia in vivo, we first established the oxaliplatin‐induced cold allodynia mice model (Figure 6b, see the Experimental Section for details). Briefly, a single dose of oxaliplatin (6 mg kg^−1^ body weight) was intraperitoneally (i.p.) injected in mice on day 0; on day 10 when cold allodynia was fully developed, we examined the effect of DeC‐1.2 by behavioral testing and calcium imaging. At the cellular level, we performed calcium imaging of the dorsal root ganglion (DRG) neurons dissociated from the mice (pointed out by arrows in white, Figure 6c). We observed that in oxaliplatin treated mice, both the amplitude of calcium influx induced by menthol activation of TRPM8 channel and the percentage of activated DRG neurons were significantly higher than those of vehicle treated mice (Figure 6d,e, traces and bars in red and green, respectively). As observed in the previous reports,^[^ ^12^, ^23^ ^]^ the activities of TRPM8 channel in the nociceptive DRG neurons were indeed up regulated by oxaliplatin to sensitize the nociceptive DRG neurons. In oxaliplatin treated mice, perfusion of DeC‐1.2 in the presence of menthol virtually abolished such increases in calcium influx in DRG neurons (Figure 6d,e, traces and bars in black, respectively). Therefore, DeC‐1.2 we designed exerted potent activity to suppress the sensitized DRG neurons in oxaliplatin treated mice.

**Figure 7:**
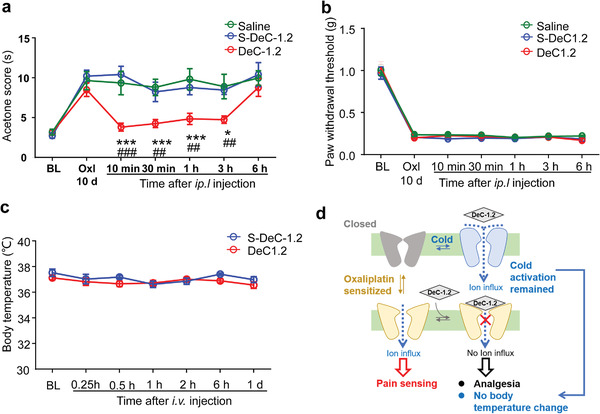
In vivo effect of DeC‐1.2 against oxaliplatin‐induced cold allodynia. a) Effect of DeC‐1.2 on oxaliplatin‐induced cold allodynia. The time spent in withdrawal, flinching, or licking the stimulated paw was recorded and blindly counted, as acetone score, during 5 min after acetone treatment. 3.5 µg of DeC‐1.2 or S‐DeC‐1.2 in 20 µL solution was injected. Data were shown as Mean ± SEM of 9–13 animals in each group. * indicates *p* < 0.05 in two‐way ANOVA. b) Effect of DeC‐1.2 on oxaliplatin‐induced mechanical allodynia. 3.5 µg of DeC‐1.2 or S‐DeC‐1.2 in 20 µL solution was injected. Data were shown as Mean ± SEM of 9–13 mice in each group. c) Effect of DeC‐1.2 on body temperature. Rectal temperatures in mice were measured before treatment (baseline, BL) and at 5 min, 15 min, 0.5, 1, 2, 6, and 24 h following DeC‐1.2 or S‐DeC‐1.2 (30 µg g^−1^, i.v.) administration. Data were shown as Mean ± SEM of 5–9 animals. N.S. indicates No significance in one‐way ANOVA. d) Schematic diagram of the modality‐specific inhibition of TRPM8 channel by DeC‐1.2.

Furthermore, DeC‐1.2 was intraplantar (i.pl.) injected to test its effect against acetone evaporation induced cooling. We found that i.pl. injection of DeC‐1.2 (3.5 µg/20 µL) into the hind‐paw plantar of oxaliplatin treated mice significantly reduced the cooling elicited pain behavior quantified by the licking, lifting, and flinching time of hind paw stimulated with acetone (acetone score), while saline or S‐DeC‐1.2 (3.5 µg/20 µL) injection exhibited no analgesic effect. Such an analgesic effect of DeC‐1.2 lasted for 3 h (**Figure** **7a**). Consistently, the magnitude of analgesic effect against cold allodynia by DeC‐1.2 application was similar to that of genetically knocking out of TRPM8 channel.^[^ ^12^ ^]^ As a negative control, we observed that in von Frey tests, while oxaliplatin treatment led to mechanical allodynia as reflected in the decrease in paw withdrawal threshold, the same i.pl. injection of DeC‐1.2 (3.5 µg/20 µL) did not change paw withdrawal threshold as compared to saline injection (Figure 7b). More importantly, we observed that intravenous (i.v.) injection of DeC‐1.2 at 30 µg g^−1^ body weight, which was high enough to largely reduce WDS behavior in icilin treated mice (Figure 6a), did not significantly alter body temperature in mice (Figure 7c). Therefore, DeC‐1.2 inhibited oxaliplatin‐induced cold allodynia in vivo without affecting body temperature as we designed.

## 3 Discussion

In this study, we have rationally designed and validated a modality‐specific cyclic peptide inhibitor of TRPM8 channel against cold pain. To alleviate the common side effects of TRP channel blockers such as altering body temperature by modality‐specific inhibition of TRPM8 channel, we chose to target the outer pore of TRPM8 channel first because this region has been a hot zone for channel blockers. For instance, Agitoxin2 and charybdotoxin bind to this region in voltage‐gated potassium channels to block ion permeation as revealed by mutant cycle analysis ^[^ ^24^ ^]^ and x‐ray crystallography,^[^ ^25^ ^]^ respectively. In TRP channels, peptide toxins targeting TRPV1 channels such as DkTx,^[^ ^26^ ^]^ RhTx,^[^ ^27^ ^]^ RhTx2,^[^ ^28^ ^]^ BmP01,^[^ ^29^ ^]^ and HCRG21^[^ ^30^ ^]^ all bind to the outer pore region.

More importantly, the outer pore of TRPM8 channel undergoes modality‐specific conformational rearrangements during channel activation, which lays the structural basis for developing modality‐specific inhibitors. In TRPV1 channel, our previous studies showed that noxious heat,^[^ ^31^ ^]^ divalent cations,^[^ ^32^ ^]^ capsaicin,^[^ ^33^ ^]^ and protons^[^ ^34^ ^]^ induce differential conformational changes in the outer pore, where the peptide toxins from sea anemone such as HCRG21, APHC1, and APHC3 bind and block the channel.^[^ ^35^ ^]^ Interestingly, by blocking TRPV1 channel pore, these toxins exert analgesic effects in mice without inducing hyperthermia.^[^ ^36^ ^]^ Moreover, second generation of small molecule TRPV1 inhibitors, which antagonize only ligand activation of the channel but not proton activation,^[^ ^37^ ^]^ are also modality‐specific so that they exert analgesic effects without altering body temperature.^[^ ^38^ ^]^ In TRPM8 channel, we have shown that cooling^[^ ^17^ ^]^ and menthol^[^ ^10^ ^]^ induce different conformational changes in the outer pore region. Therefore, as designed by our OHCA stratagem to target the outer pore in TRPM8, our DeC‐1.2 exhibited modality‐specific activities, where only the ligand activation but not the cold activation of the channel was inhibited (Figure 3), so that DeC‐1.2 did not alter body temperature while exerting analgesic effects in mice (Figure 7). Therefore, we believe that differences in channel protein structural changes are the prerequisite for developing a modality‐specific inhibitor, and rational protein design methods such as our OHCA are needed to make a peptide binding to a specific conformation of the target protein.

DeC‐1.2 offered novel insights regarding mechanisms of chemotherapy‐induced neuropathy. As our modality‐specific DeC‐1.2 relieved the oxaliplatin‐induced cold allodynia, it suggested that besides the increase in TRPM8 expression,^[^ ^39^ ^]^ ligand‐induced activities of TRPM8 channel were sensitized by oxaliplatin. TRP channels including TRPM8 are polymodal receptors, so different activation modalities of these channels are allosterically coupled.^[^ ^40^ ^]^ Sensitized ligand activation of TRPM8 likely by endogenous agonists would enhance temperature activation of this channel, so that normal coolness may elicit a larger current response to incur cold allodynia.

Peptides in general exhibit high affinity and selectivity for their target despite difficulties in drug delivery. For instance, the FDA‐approved peptide analgesic drug ziconotide exerts its effects by potent and selective blocking Ca_V_2.2 channel.^[^ ^41^, ^42^ ^]^ Ziconotide is delivered by intrathecal injection in patient with severe chronic pain.^[^ ^41^ ^]^ In this case, our rational design has created DeC‐1.2 molecule that exhibits nanomolar IC_50_ and high selectivity for TRPM8 channel, thus further antagonizes cooling‐induced pain in oxaliplatin treated mice. Though the analgesic effects of DeC‐1.2 lasted for about 3 h, as the peptide may be degraded in vivo, with advances in peptide packing and delivery techniques,^[^ ^43^ ^]^ peptides are gaining momentum in analgesic drug development.^[^ ^44^ ^]^ Therefore, DeC‐1.2 forms the basis for further drug development treating chemotherapy‐induced neuropathic pain, such as the cold allodynia induced by oxaliplatin.

## 4 Experimental Section

### Molecular Design

The outer pore region of mouse TRPM8 in RosettaCM^[^ ^45^ ^]^ was first homology modeled using the cryo‐EM structure of TRPM4 (PDB ID: 6BCL) as template. A total of 10 000 models were generated and top 10 model of the total score were well converged. The model with the lowest total score was chosen as the model for peptide design.

Peptide binders to the TRPM8 channel outer pore region were de novo designed following the OHCA used to design the peptidic positive allosteric modulator of TRPV1 channel^[^ ^20^ ^]^ and described previously.^[^ ^18^, ^20^ ^]^ Briefly, each of the natural amino acids was docked to outer pore in Rosetta.^[^ ^46^ ^]^ One arginine residues bound well to the outer pore so that it was chosen as the hotspot for subsequent protein design. The inverse rotamer library of this arginine residue was generated using RosettaScripts^[^ ^47^ ^]^ (Script S1, Supporting Information). The scaffold library was generated by selecting protein structures from the PDB database with the following criteria:

1. There is not any DNA, RNA, or disulfide bond.
2. There is only one protein chain; stoichiometry is monomer.
3. There are less than 40 residues.
4. There is no ligand presented in structure.
5. Homologue removal is set at 70% identity.

They were further cleaned and prepacked in Rosetta (Script S2 and S3, Supporting Information). The outer pore was then docked to the scaffold library in a coarse‐grained manner with the PatchDock software^[^ ^48^ ^]^ based on protein structure shape complementarity. The patchdocked scaffolds were fused with the hotspot, and then the protein–protein interface was redesigned by RosettaScripts (Script S4, Supporting Information). These initial designs were again screened. Only the designs with shape complementarity, ddg and SASA larger than 0.6, −20 Rosetta Energy Unit (REU) and 500 Å^2^, respectively, were kept. The design with the largest *ddg* and *score* were selected as the final design. The designs were further subjected to computational affinity maturation in Rosetta (Script S5, Supporting Information).

All the molecular graphics of protein structure models were rendered by UCSF Chimera^[^ ^49^ ^]^ software version 1.12.

### Designed Peptides Synthesis and Purification

Designed cyclic peptides with a disulfide bridge were synthesized by GL Biochem (Shanghai) Ltd. Crude peptides were further purified by reverse‐phase (RP) HPLC. Once the purity of a peptide of interest was determined to be higher than 95% by HPLC chromatography and MALDI‐TOF mass spectrometry or electrospray ionization mass spectrometry (ESI‐MS), the peptide peaks in HPLC were pooled and lyophilized.

### Cell Culture, Transient Transfection

HEK293T cells were cultured in Dulbecco's modified Eagle's medium with 10% fetal bovine serum, penicillin (100 U mL^−1^), and streptomycin (100 mg mL^−1^) at 37 °C with 5% CO_2_. Transient transfection was conducted by using Lipofectamine 2000 (Invitrogen) and following the instruction manual.

### Electrophysiology

Electrophysiological experiments were performed between 24 and 48 h after transfections. Specifically, single‐channel recordings were conducted about 8 h after transfection.

Patch‐clamp recordings were performed with a HEKA EPC10 amplifier with PatchMaster software (HEKA) in the whole‐cell, inside‐out, or outside‐out configuration. For whole cell recordings, patch pipettes were prepared from borosilicate glass and fire‐polished to a resistance of ≈3–6 MΩ. Serial resistance was compensated by 60%. Whole‐cell recordings were performed at ±80 mV. For single‐channel recordings, patch pipettes were fire‐polished to a higher resistance of 6–8 MΩ. Current signal was sampled at 10 kHz and filtered at 2.9 kHz. Membrane potential was clamped at +80 mV. All recordings were performed at room temperature (22 °C) with the maximum variation of 1 °C.

There were different solutions prepared for different ion channels current recordings. For TRPM8, TRPV1, TRPV2, TRPV3, and TRPA1 channels recording, the standard bath and pipette solution contained: 130 × 10^−3^ m NaCl, 0.2 × 10^−3^ m EDTA, and 3 × 10^−3^ m HEPES, pH = 7.2. For TRPM4 channels recording, solutions with 130 × 10^−3^ m NaCl and 3 × 10^−3^ m HEPES, pH = 7.2 were used as standard bath solution. The pipette solutions required 500 × 10^−6^ m CaCl_2_ mixing with 130 × 10^−3^ m NaCl and 3 × 10^−3^ m HEPES, pH = 7.2. TRPM2 currents were recorded with the help of standard bath solution of 147 × 10^−3^ m NaCl, 2 × 10^−3^ m KCl, 1 × 10^−3^ m MgCl_2_, 10 × 10^−3^ m HEPES, 2 × 10^−3^ m CaCl_2_, 13 × 10^−3^ m glucose, pH = 7.4 and the pipette solutions of 147 × 10^−3^ m NaCl, 1 × 10^−3^ m MgCl_2_, 10 × 10^−3^ m HEPES, pH = 7.4. For Na_V_1.5, Na_V_1.7 channels, the standard bath solution was 140 × 10^−3^ m NaCl, 3 × 10^−3^ m KCl, 1 × 10^−3^ m MgCl_2_, 1 × 10^−3^ m CaCl_2_, 10 × 10^−3^ m HEPES, pH = 7.2 and standard pipette solution was 140 × 10^−3^ m CsF, 1 × 10^−3^ m EGTA, 10× 10^−3^ m NaCl, 10 × 10^−3^ m MgCl_2_, 3 × 10^−3^ m KCl, pH = 7.2. To evoke sodium channel currents, a holding potential of −80 mV was used with a testing pulse to +10 mV.

To apply solutions containing specific reagents during patch‐clamp recording, a rapid solution changer with a gravity‐driven perfusion system was used (RSC‐200, BioLogic). Each solution was delivered through a separate tube so that there was no mixing of solutions. Pipette tip with a membrane patch was placed directly in front of the perfusion outlet during the recordings.

### Animal Test—Animals

Adult CD1 mice (male, 6–8 weeks) were used for behavioral and primary cultures of DRG neurons studies. Mice were group‐housed under the 12 h light‐dark cycle with access to standard food and water ad libitum. All the animal procedures were approved by the Institutional Animal Care & Use Committee (IACUC) of Zhejiang University.

### Animal Models of Pain

Animals were habituated to the testing environment for at least 2 d before testing. i.p. injection of oxaliplatin (6 mg kg^−1^ for a single injection) was given to generate chemotherapy‐induced neuropathic pain. Neuropathic pain behaviors were tested about 10 d after oxaliplatin injection.

### Icilin‐Induced Wet‐Dog Shake Test

Six to nine adult CD1 male mice were prepared for experimental or control groups. The animals were pretreated with DeC‐1.2 or vehicle via i.v. at 30 min before icilin (i.p., 2.5 µg g^−1^ body weight) treatment. Wet dog shake behavior was recorded and counted for 30 min following icilin administration.

### Body Temperature Measurement

A digital thermometer (FT3400) was used for body temperature measurement. Adult CD1 male mice were placed into an environmental room maintained at a constant temperature of 22 ± 1 °C. Body temperature was recorded using a rectal probe for mice (FT3400), which was inserted ≈2 cm from the anus. Body temperature was measured before treatment (baseline, BL) and at 5 min, 15 min, 0.5,1,2, 6, and 24 h following DeC‐1.2 (30 mg kg^−1^, i.v.) administration.

### Oxaliplatin‐Induced Cold Allodynia

Cold allodynia was performed via acetone‐induced evaporating cold. 50 µL acetone was gently splashed to the hind‐paw plantar of oxaliplatin‐treated mice via a syringe attached with plastic tube. The time spent in withdrawal, flinching or licking the stimulated paw was recorded and blindly counted during 5 min after acetone treatment. 3.5 µg/20 µL DeC‐1.2 or vehicle was intraplantarly injected to assess its analgesic effect on oxaliplatin‐induced allodynia.

### Oxaliplatin‐Induced Mechanical Allodynia

Mechanical allodynia was assessed by measuring paw withdrawal thresholds in von Frey test. To determine mechanical thresholds of paw withdrawal, a series of von Frey hairs (0.02–2.56 g, Stoelting) with increasing stiffness were used according to Dixon's up‐down method. The von Frey test was performed at pre‐ or post‐treatment with oxaliplatin or DeC‐1.2. 3.5 µg/20 µL DeC‐1.2 was intraplantarly injected to assess its analgesic effect on oxaliplatin‐induced allodynia.

### Calcium Imaging of Mice DRG Neurons

Mouse DRGs were aseptically dissected from vehicle‐treated mice and oxaliplatin induced neuropathic pain model mice and digested with collagenase (0.2 mg mL^−1^, Roche)/dispase‐II (3 mg mL^−1^, Roche) for 1 h. Then, single‐cell suspension was prepared via mechanical trituration and centrifugation. Cells were placed on glass coverslips coated with poly‐d‐lysine and cultured with the neurobasal medium containing 2% B27 supplement, 1 × 10^−3^ m l‐glutamine, 50 ng mL^−1^ NGF 2.5s, 50 units mL^−1^ penicillin, and 50 µg mL^−1^ streptomycin. Primary‐cultured DRG neurons were maintained at 37 °C incubator with 5% CO_2_ for 24 h before experiments.

Extracellular solution (ECS) was 140 × 10^−3^ m NaCl, 5 × 10^−3^ m KCl, 1 × 10^−3^ m MgCl_2_, 1.8 × 10^−3^ m CaCl_2_, 10 × 10^−3^ m glucose, 15 × 10^−3^ m HEPES, pH = 7.4. DRG neurons were washed twice with an ECS, followed by incubation in 2 mL of ECS supplemented with 2 × 10^−6^ m Fluo‐2 AM and 0.05% Pluronic F‐127 (both from Molecular Probes) at 37 °C for 30 min. The cell images were captured via a cooled Digital CMOS camera (ORCA‐Flash 4.0, Hamamatsu Photonics K.K, Japan) under the excitation wavelength of 340 and 380 nm. The fluorescence intensity ratio 340/380 in each experiment was analyzed using Visiview software (Visitron System GmbH). Menthol (100 × 10^−6^ m) premixed with or without DeC‐1.2 (10 × 10^−6^ m) was applied for 30 s via the fast exchange perfusion system (ALA‐VM8; ALA Scientific Instruments). Menthol‐induced iCa^2+^ mobilization was analyzed via ratio change of fluorescence intensity 340/380 and normalized to the baseline ratio of 340/380 nm. The effect of DeC‐1.2 on menthol‐induced iCa^2+^ signal was evaluated via the percentage of responsive neurons and the maximum of ratio change 340/380 in responsive neurons.

*Statistics*: All experiments were independently repeated for at least three times. All statistical data are given as Mean ± Sem. Electrophysiology data were analyzed using paired or unpaired Student's *t*‐test. Behavioral data were analyzed using Student's *t*‐test, one‐way or two‐way ANOVA. N.S. indicates no significance. *, **, ***, and **** indicate *p* < 0.05, *p* < 0.01, *p* < 0.001, and *p* < 0.0001, respectively.

## Conflict of Interest

The authors declare no conflict of interest.

## Author Contributions

A.A., Y.‐K.X., and W.D. contributed equally to this work. A.A., Y.K.X., W.D., Z.H.Y., A.A, J.J.L., and Z.Z. conducted the experiments including patch‐clamp recordings, peptide purification, and animal behavior tests; F.Y. designed the protein modulators; S.Y., Z.Z.X., and F.Y. conceived and supervised the project and prepared the manuscript; S.Y., Z.Z.X., and F.Y. participated in data analysis and manuscript writing.

## Supporting information

Supporting Information

## Data Availability

All data needed to evaluate the conclusions in the paper are present in the paper and/or the Supporting Information. Additional data available from authors upon request.
