# Supplementary material for: Rational Design of a Modality‐Specific Inhibitor of TRPM8 Channel against Oxaliplatin‐Induced Cold Allodynia
Source: Adv Sci (Weinh). 2021 Oct 17;8(22):2101717. doi: 10.1002/advs.202101717 (PMC8596132; doi:10.1002/advs.202101717)
Supplement: Supplementary file 1 — Supporting Information [file ADVS-8-2101717-s001.pdf]

© 2021 Wiley-VCH GmbH

ADVANCED SCIENCE
Open Access

Supporting Information

for *Adv. Sci.*, DOI: 10.1002/advs.202101717

Rational design of a modality-specific inhibitor of TRPM8 channel against oxaliplatin-induced cold allodynia

Aerziguli Aierken[1,3,5] *Ya-kai Xie*[2,3,5] *Wenqi Dong*[4,5] *Abuliken Apaer*[1] *Jia-Jia Lin*[2,3] *Zihan Zhao*[4] *Shilong Yang*[4*] *Zhen-Zhong Xu*[2,3*] *Fan Yang*[1,3*]

Rational design of a modality-specific inhibitor of TRPM8 channel against oxaliplatin-induced cold allodynia

Aerziguli Aierken[1,3,5], Ya-kai Xie[2,3,5], Wenqi Dong[4,5], Abuliken Apaer[1], Jia-Jia Lin[2,3] Zihan Zhao[4], Shilong Yang[4]*, Zhen-Zhong Xu[2,3]*, Fan Yang[1,3]*

[1] Department of Biophysics, and Kidney Disease Center of the First Affiliated Hospital, Zhejiang University School of Medicine, 310058 Hangzhou, Zhejiang Province, China
[2] Department of Neurobiology and Department of Anesthesiology of First Affiliated Hospital, Zhejiang University School of Medicine, Hangzhou, Zhejiang 310058, China
[3] NHC and CAMS Key Laboratory of Medical Neurobiology, MOE Frontier Science Center for Brain Research and Brain–Machine Integration, School of Brain Science and Brain Medicine, Zhejiang University, Hangzhou, Zhejiang 310058, China
[4] College of Wildlife and Protected Area, Northeast Forestry University, Harbin, 150040, China
[5] These authors contributed equally to this work
*Correspondence should be addressed to:
Fan Yang (fanyanga@zju.edu.cn), Zhen-Zhong Xu (xuzz@zju.edu.cn) or Shilong Yang (syang2020@nefu.edu.cn)

SUPPLEMENTARY FIGURE LEGENDS

Figure S1. HPLC and MS report of DeC peptides. (a) The purity of DeC-1.1 (where the arrow pointed) was determined by RP-HPLC higher than 95%. (b) The molecular weight of DeC-1.1 was determined by MALDI-TOF to be1473.68 Da. (c) The purity of DeC-1.2 (where the arrow pointed to) was determined by RP-HPLC to be higher than 95%. (d) The molecular weight of DeC-1.2 was determined by MALDI-TOF to be 1774.37 Da, which is virtually identical to its theoretical molecular weight (1774.03 Da).

Figure S2. HPLC and MS reports of DeC-1.2 mutants in alanine scan. (a) HPLC and MS report of DeC-1.2 R2A. The purity of DeC-1.2 R2A was determined by RP-HPLC higher than 95%. The molecular weight of DeC-1.2 R2A was determined by ESI to be1688.89 Da. (b) HPLC and MS report of DeC-1.2 R3A. The purity of DeC-1.2 R3A was determined by RP-HPLC higher than 95%. The molecular weight of DeC-1.2 R3A was determined by ESI to be1688.89 Da. (c) HPLC and MS report of DeC-1.2 D4A. The purity of DeC-1.2 A was determined by RP-HPLC higher than 95%. The molecular weight of DeC-1.2 D4A was determined by ESI to be 1729.99 Da.(d) HPLC and MS report of DeC-1.2 R5A. The purity of DeC-1.2 A was determined by RP-HPLC higher than 95%. The molecular weight of DeC-1.2 R5A was determined by ESI to be 1688.89 Da.(e) HPLC and MS report of DeC-1.2 R7A. The purity of DeC-1.2 R7A was determined by RP-HPLC higher than 95%. The molecular weight of DeC-1.2 R7A was determined by ESI to be 1688.89 Da. (f) HPLC and MS report of DeC-1.2 H8A. The purity of DeC-1.2 A was determined by RP-HPLC higher than 95%. The molecular

weight of DeC-1.2 H8A was determined by ESI to be 1707.94 Da. (g) HPLC and MS report of DeC-1.2 Y9A. The purity of DeC-1.2 A was determined by RP-HPLC higher than 95%. The molecular weight of DeC-1.2 Y9A was determined by ESI to be 1681.91 Da. (h) HPLC and MS report of DeC-1.2 R10A. The purity of DeC-1.2 R10A was determined by RP-HPLC higher than 95%. The molecular weight of DeC-1.2 R10A was determined by ESI to be 1688.89 Da. (i) HPLC and MS report of DeC-1.2 Q11A. The purity of DeC-1.2 Q11A was determined by RP-HPLC higher than 95%. The molecular weight of DeC-1.2 Q11A was determined by ESI to be 1716.95 Da. (j) HPLC and MS report of DeC-1.2 R12A. The purity of DeC-1.2 R12A was determined by RP-HPLC higher than 95%. The molecular weight of DeC-1.2 R12A was determined by ESI to be 1688.89 Da.

Figure S3. HPLC and MS reports of DeC-1.2 mutants as compared to DeC-1.1 and S-DeC-1.2. (a) HPLC and MS report of DeC-1.2 R3N. The purity of DeC-1.2 R3N was determined by RP-HPLC higher than 95%. The molecular weight of DeC-1.2 R3N was determined by ESI to be1731.93 Da. (b) HPLC and MS report of DeC-1.2 D4S. The purity of DeC-1.2 D4S was determined by RP-HPLC higher than 95%. The molecular weight of DeC-1.2 D4S was determined by ESI to be1861.09 Da. (c) HPLC and MS report of DeC-1.2 Y9D. The purity of DeC-1.2 Y9D was determined by RP-HPLC higher than 95%. The molecular weight of DeC-1.2 Y9D was determined by ESI to be 1725.93 Da.(d) HPLC and MS report of DeC-1.2 R10S. The purity of DeC-1.2 R10S was determined by RP-HPLC higher than 95%. The molecular weight of DeC-1.2 R10S was determined by ESI to be 1704.91Da.(e) HPLC and MS report of DeC-1.2 R12K. The purity of DeC-1.2 R12K was determined by RP-HPLC higher than 95%. The molecular weight of DeC-1.2 R12K was determined by ESI to be 1902.19Da. (e) HPLC and MS report of S-DeC-1.2. The purity of S-DeC-1.2 was determined by RP-HPLC higher than 95%. The molecular weight of S-DeC-1.2 was determined by ESI to be 1774.03Da.

Supplementary table 1. IC_{50} values of DeC-1.2 inhibition on TRP channels and Sodium channels.

Supplementary table 2. The amino acid sequences of DeC peptides for alanine scan.

Supplementary table 3. The amino acid sequences of DeC-1.2 and its mutants as compared to DeC-1.1.

SUPPLEMENTARY SCRIPTS

Supplementary Script 1. Building the inverse rotamer library.

```
-database /home/fan/rosetta_2016.20/main/database
-ignore_unrecognized_res
-in:file:fullatom
-ex1
-ex2
#-in:file:s
/media/fan/Data/Rosetta/Project_TRPM8_design/dock_AA/Arg/ForIR_ini_Arg_4D_1
D_SFonly_mTRPM8_0674.pdb
-in:file:s
/media/fan/Data/Rosetta/Project_TRPM8_design/dock_AA/Arg/r_ForIR_Arg.pdb
-parser:protocol
/media/fan/Data/Rosetta/Project_TRPM8_design/dock_AA/Arg/InverseRotamers_fy
.xml
-nstruct 10000
-overwrite
```

InverseRotamers_fy.xml:

```
<dock_design>
    <SCOREFXNS>
        <stub_dock weights=talaris2014 />
    </SCOREFXNS>
    <FILTERS>
        <EnergyPerResidue name=energy scorefxn=stub_dock pdb_num=1B
energy_cutoff=1/>
        <Ddg name=ddg scorefxn=stub_dock threshold=-1 repack=0/>
    </FILTERS>
    <MOVERS>
        <TryRotamers name=try pdb_num=1B /> list residues the backbones of
which are to be ignored in energy evaluations under the shove flag
        RepackMinimize name=rpk repack_partner1=1 repack_partner2=0
design_partner1=0 design_partner2=0 minimize_bb=0 minimize_rb=0
minimize_sc=1
    </MOVERS>
    <APPLY_TO_POSE>
    </APPLY_TO_POSE>
    <PROTOCOLS>
        <Add mover_name=try/>
        add mover_name=rpk
        <Add filter_name=energy/>
        <Add filter_name=ddg/>
    </PROTOCOLS>
</dock_design>
```

Supplementary Script 2. Clean of the protein structures selected from PDB database.

```
#!/bin/bash
FOLDER_A=/media/Data_Ubuntu/PatchDock/scaffold
FOLDER_B=/media/Data_Ubuntu/PatchDock/scaffold_cleaned

for file_a in ${FOLDER_A}/*; do
    file_a_prefix=${file_a%.*}
    file_prefix=${file_a_prefix#${FOLDER_A}/*}
    echo $file_prefix
    sudo cleanPdb.pl -pdbfile ${FOLDER_A}/$file_prefix.pdb >
${FOLDER_B}/$file_prefix.pdb
done
```

Supplementary Script 3. Prepack of the cleaned protein structures selected from PDB database.

```
#!/bin/bash
FOLDER_A=/media/Data_Ubuntu/PatchDock/scaffold_cleaned

for file_a in ${FOLDER_A}/*; do
    file_a_prefix=${file_a%.*}
    file_prefix=${file_a_prefix#${FOLDER_A}/*}
    echo $file_prefix
    /home/fan/rosetta_source/bin/rosetta_scripts.linuxgccrelease -ex1 -
ex2aro -database /home/fan/rosetta_source/rosetta_database -
ignore_unrecognized_res -in:file:fullatom -in:file:s
/home/fan/PatchDock/PDB/scaffold_PDB_1/$file_prefix.pdb -parser:protocol
/home/fan/rosetta_source/input_files/prepack/ppk.xml -overwrite -out:output
    sudo mv $file_prefix*.pdb $file_prefix.pdb
    sudo cp $file_prefix.pdb
/media/Data_Ubuntu/PatchDock/scaffold_cleaned_prepacked/
    rm -r $file_prefix.pdb
done
```

Supplementary Script 4. Fusion of scaffold proteins with the hotspots.

```
#!/bin/bash
#$ -S /bin/bash
#$ -e /home/fanyang/work/Placestub/$TASK_ID/
#$ -o /home/fanyang/work/Placestub/$TASK_ID/

n=(${SGE_TASK_ID}-1)*100+1
for ((i=1; i<101; i=i+1))
do /home/fanyang/rosetta_source/bin/rosetta_scripts.static.linuxgccrelease
\
-in:path:database /home/fanyang/rosetta_database \
-in:file:fullatom \
-ex1 \
-ex2 \
-in:file:s /home/fanyang/projects/input_files/Docked/$[${i}+${n}].pdb \
-out:file:scorefile mscore_$[${i}+${n}].fsc \
-nstruct 50 \
-out:file:silent /home/fanyang/work/Placestub/${SGE_TASK_ID}/
placestubs_${SGE_TASK_ID}.silent \
-out:file:silent_struct_type binary \
-mute all \
-parser:protocol
/home/fanyang/projects/input_files/OneResidueHotspot_fy.xml
done
```

OneResidueHotspot_fy.xml:

```
<dock_design>
    <TASKOPERATIONS>
        PreventRepacking name=prevent_repacking_R913 resnum=913

    </TASKOPERATIONS>
    <SCOREFXNS>
        <stub_docking_low weights=interchain_cen hs_hash=10.0/>
        <ddg_scorefxn weights=talaris2014.wts patch=talaris2014.wts
hs_hash=0.0/>
        <score12_coordcst weights=talaris2014.wts patch=talaris2014.wts
hs_hash=0.0>
            <Reweight scoretype=coordinate_constraint weight=1.0/>
        </score12_coordcst>
  </SCOREFXNS>
   <FILTERS>
         <Ddg name=ddg threshold=-10 scorefxn=ddg_scorefxn repeats=3/>
         <Sasa name=sasa threshold=600/>
```

```
        <CompoundStatement name=ddg_sasa> for the loop over filter
            <AND filter_name=ddg/>
            <AND filter_name=sasa/>
        </CompoundStatement>
        TerminusDistance name=termini distance=0
    AtomicContact name=touch_345 residue1=349A distance=5.2 W on target
        AtomicContact name=touch_365 residue1=369A distance=6.5 T near W
        CompoundStatement name=touch_TW
            AND filter_name=touch_345
            AND filter_name=touch_365
        CompoundStatement>
  </FILTERS>
  <MOVERS>
    <Docking name=dock fullatom=0 local_refine=0
score_low=stub_docking_low/>
        <RepackMinimize name=des1 minimize_bb=0 minimize_rb=1
scorefxn_repack=soft_rep scorefxn_minimize=score_docking
interface_cutoff_distance=10.0/>
        <RepackMinimize name=des2 minimize_bb=0 minimize_rb=1
interface_cutoff_distance=10.0/>
        <RepackMinimize name=des3 minimize_bb=0 minimize_rb=1
interface_cutoff_distance=10.0/>
        <SaveAndRetrieveSidechains name=srsc/>
        <BackrubDD name=br interface_distance_cutoff=10.0
bbg_move_probability=0.0 small_move_probability=0.0/>

        <PlaceStub name=place_arg place_scaffold=1 chain_to_design=2
stubfile="/media/fan/Data/Rosetta/Project_TRPM8_design/InverseRotamers/IR_A
rg/IR_Arg_TRPM8.pdb" add_constraints=1 minimize_rb=1 hurry=1
score_threshold=300.0 stub_energy_threshold=300.0 max_cb_dist=4.0
leave_coord_csts=1 final_filter=ddg_sasa> PlaceStub's task operations will
be fed down to all movers that are mentioned as task aware.
leave_coord_csts lets downstream movers use the coordinate csts that
placestub found to be useful
            <StubMinimize>
                <Add mover_name=br bb_cst_weight=20/>
            </StubMinimize>
            <DesignMovers>
                Add coord_cst_std=1.0
                <Add mover_name=srsc/>
                <Add mover_name=des1 coord_cst_std=1.0/>
                <Add mover_name=des2 coord_cst_std=1.5/>
                <Add mover_name=br/>
                <Add mover_name=des3/>
```

```
                </DesignMovers>
                </PlaceStub>
            </MOVERS>
        <APPLY_TO_POSE>
            <SetupHotspotConstraints
stubfile="/media/fan/Data/Rosetta/Project_TRPM8_design/InverseRotamers/IR_A
rg/IR_Arg_TRPM8.pdb" cb_force=0.5/>
                profile weight=0.2
file_name="/media/Data_Ubuntu/PatchDock/Docked_1/%%pdb_name%%.cst"
        </APPLY_TO_POSE>
        <PROTOCOLS>
             <Add mover_name=dock />
                <Add mover_name=place_arg/>
                <Add filter_name=ddg/>
                <Add filter_name=sasa/>
        </PROTOCOLS>
</dock_design>
```

Supplementary Script 5. In silico affinity maturation of candidate designes.

```
/home/fanyang/rosetta_source/bin/rosetta_scripts.static.linuxgccrelease
-database /home/fan/rosetta_source/rosetta_database
-ignore_unrecognized_res
-in:file:fullatom
-ex1
-ex2
-in:file:s /media/Data2/Academic/Rosetta/initial_design.pdb
-parser:protocol
/media/Data2/Academic/Rosetta/Rosetta_script/xml_scripts_Fan/Multicriterion
Optimization_fy.xml
-nstruct 1000
-overwrite
```

MulticriterionOptimization_fy.xml:

```
<dock_design>
        <TASKOPERATIONS>
                <InitializeFromCommandline name=init/>
                <ProteinInterfaceDesign name=pido
interface_distance_cutoff=10/>
                <RestrictAbsentCanonicalAAS name=nohis
keep_aas="ACDEFGIKLMNPQRSTVWY"/>
        </TASKOPERATIONS>
        <SCOREFXNS>
        </SCOREFXNS>
        <FILTERS>
```

```
                <Sasa name=hydrophobic_sasa hydrophobic=1 confidence=0/>
                <Sasa name=sasa confidence=0/>
                <Ddg name=ddg confidence=0 repeats=3/>
                <ScoreType name=total_score score_type=total_score
threshold=0/>
        </FILTERS>
        <MOVERS>
                <AtomTree name=docking_tree docking_ft=1/>
                <RandomMutation name=random_mutation
task_operations=init,pido,restrict256,restrict260,nohis/>
                <MinMover name=min bb=1 chi=1 jump=1>
                        <MoveMap>
                                <Chain number=1 chi=1 bb=0/>
                        </MoveMap>
                </MinMover>
                <ParsedProtocol name=agg_mover>
                        <Add mover=random_mutation/>
                        <Add mover=min/>
                </ParsedProtocol>
                <GenericMonteCarlo name=genericMC mover_name=agg_mover
filter_name=ddg temperature=0.1 trials=1000>
                        <Filters>
                                <AND filter_name=total_score
temperature=1/>
                        </Filters>
                </GenericMonteCarlo>
        </MOVERS>
        <APPLY_TO_POSE>
        </APPLY_TO_POSE>
        <PROTOCOLS>
                <Add mover=docking_tree/>
                <Add mover=genericMC/>
                <Add filter=ddg/>
                <Add filter=sasa/>
        </PROTOCOLS>
</dock_design>
```
